# The Dutch version of the knee injury and osteoarthritis outcome score: A validation study

**DOI:** 10.1186/1477-7525-6-16

**Published:** 2008-02-26

**Authors:** Ingrid B de Groot, Marein M Favejee, Max Reijman, Jan AN Verhaar, Caroline B Terwee

**Affiliations:** 1 Department of Orthopaedics, Erasmus University Medical Center, Rotterdam, The Netherlands; 2 EMGO Institute, VU University Medical Center, Amsterdam, The Netherlands

## Abstract

**Background:**

The Knee Injury and Osteoarthritis Outcome Score (KOOS) was constructed in Sweden. This questionnaire has proved to be valid for several orthopedic interventions of the knee. It has been formally translated and validated in several languages, but not yet in Dutch. The purpose of the present study was to evaluate the clinimetric properties of the Dutch version of the KOOS questionnaire in knee patients with various stages of osteoarthritis (OA).

**Methods:**

The Swedish version of the KOOS questionnaire was first translated into Dutch according to a standardized procedure and second tested for clinimetric quality. The study population consisted of patients with different stages of OA (mild, moderate and severe) and of patients after primary TKA, and after a revision of the TKA. All patients filled in the Dutch KOOS questionnaire, as well as the SF-36 and a Visual Analogue Scale for pain. The following analyses were performed to evaluate the clinimetric quality of the KOOS: Cronbach's alpha (internal consistency), principal component analyses (factor analysis), intraclass correlation coefficients (reliability), spearman's correlation coefficient (construct validity), and floor and ceiling effects.

**Results:**

For all patients groups Cronbach's alpha was for all subscales above 0.70. The ICCs, assessed for the patient groups with mild and moderate OA and after revision of the TKA patients, were above 0.70 for all subscales. Of the predefined hypotheses 60% or more could be confirmed for the patients with mild and moderate OA and for the TKA patients. For the other patient groups less than 45% could be confirmed. Ceiling effects were present in the mild OA group for the subscales Pain, Symptoms and ADL and for the subscale Sport/Recreation in the severe OA group. Floor effects were found for the subscales Sport/Recreation and Qol in the severe OA and revision TKA groups.

**Conclusion:**

Based on these different clinimetric properties within the present study we conclude that the KOOS questionnaire seems to be suitable for patients with mild and moderate OA and for patients with a primary TKA. The Dutch version of the KOOS had a lower construct validity for patients with severe OA on a waiting list for TKA and patients after revision of a TKA. Further validation studies on the Dutch version of the KOOS should also include a knee specific questionnaire for assessing the construct validity.

## Introduction

There is consensus that patient-reported outcomes have additional value to clinical variables to evaluate patients' health. The underlying principle is that functional status and quality of life can better be described by the patients themselves than by a physician [1]. With regards to knee surgery, however, at the start of the present study almost no reliable and validated Dutch versions of disease-specific questionnaires were available to evaluate the functional status of patients and quality of life after surgery. The Western Ontario and McMaster Universities Osteoarthritis Index (WOMAC) is recommended for the assessment of treatment effects in patients with osteoarthritis (OA) and was developed for elderly with OA and assesses pain, stiffness and function in daily living [2-4].

Traumatic knee injuries often cause damage to structures such as ligaments, menisci and cartilage, and may lead to early development of OA. To be able to follow patients after a trauma and to monitor the changes in functional status and quality of life over time, a questionnaire is needed which covers both the short and long-term consequences of an injury of the knee [5]. In other words, there is a clear need for an instrument that not only monitors the outcome in elderly knee OA patients, but also monitors the consequences of acute knee injury in physically active patients in their early adulthood.

Therefore, Roos et al. developed such a questionnaire in Sweden [6,7]. The Knee Injury and Osteoarthritis Outcome Score (KOOS) evaluates the functional status and quality of life of patients with any type of knee injury who are at increased risk to develop OA; i.e. patients with anterior cruciate ligament (ACL) injury, meniscus injury or chondral injury. Until now, the KOOS questionnaire has been validated for several orthopedic interventions such as ACL reconstruction [7], total knee arthroplasty (TKA) [8], and menisectomy [9]. It has been formally translated and validated in several languages, but not yet in Dutch.

The purpose of this study was therefore to translate the KOOS questionnaire into Dutch and to evaluate the clinimetric properties of the Dutch version of the KOOS questionnaire, in terms of internal consistency, reliability, validity, and floor and ceiling effects.

We studied the Dutch version of the KOOS in patients with different stages of OA: mild, moderate and severe OA and in patients after a primary TKA and after revision of the TKA.

## Methods

The study was divided into two stages. First, the Swedish version of the KOOS questionnaire was translated into Dutch according to a standardized procedure [10]. Second, the translated version was tested for clinimetric quality in a prospective study.

### Procedure of translation

The procedure of translation included three steps [11]. First two persons (T1 and T2) translated independently of each other the Swedish version of the KOOS questionnaire into Dutch (forward translation); one translator had a technical background and the other had a medical background; both were native Dutch speakers. Based on a consensus meeting one final version (T-12) was formed [10].

Second, two bilingual persons (T3 and T4), one with a background in education and the other with a chemical background, both native Swedish speakers, independently re-translated this Dutch version (T-12) into Swedish (backward translation). They were blind to the original Swedish version.

Finally, all translators had a consensus meeting to consolidate the final version of the Dutch version of the KOOS questionnaire, which was used in the present study. This final version was presented to a subset of 15 patients suffering from knee complaints. These patients were asked whether they understood all items and whether they had any problems with the formulation of the items on the Dutch version of the KOOS questionnaire. None of the patients reported problems with the items of the KOOS questionnaire.

### Patients

We used five patient groups with different stages of OA of the knee of the knee, based on clinical and radiographic signs, to evaluate the clinimetric properties of the Dutch version of the KOOS questionnaire. All patients were under medical treatment at the department of Orthopedics at the Erasmus Medical Center in Rotterdam between 1990 and 2005.

The first patient group consisted of patients with mild OA, who had undergone ACL reconstruction between 1994 and 1996. The second patient group consisted of patients with moderate OA who had undergone HTO between 1998 and 2000. All patients in this group had a valgus correction within a range of 5 to 14 degrees. The third patient group consisted of patients with severe OA who were on the waiting list for a TKA. The fourth patient group consisted of patients 6 months after a TKA, who were operated between 2004 and 2006. The fifth patient group consisted of patients who had undergone a revision of the primary TKA because of a failure of the primary TKA between 2001 and 2006. Patients unable to understand Dutch written language were excluded. The Medical Ethics Committee at the Erasmus Medical Center approved all studies. The choice of our study population, except for the TKA population, was based on existing retrospective cohort studies.

All participants were asked to complete three questionnaires at home: the Dutch KOOS, the SF-36 [12], and a Visual Analogue Scale for pain [13] between June 2004 and July 2006. They were asked to fill in the Dutch KOOS at home again after two till three weeks. For test-retest studies the time interval needs to be sufficiently short to support the assumption that the patients remain stable and to be sufficiently long to prevent recall [14]. We considered a time interval of three weeks to be appropriate for these patient populations. The local Medical Ethics Committee approved the study and all participants gave their written informed consent.

#### Questionnaires

##### KOOS

The KOOS questionnaire covers five dimensions that are reported separately: Pain (nine items), Symptoms (seven items), activities of daily living (ADL, 17 items), sport and function (Sport/recreation, five items), and knee-related quality of life (QoL, four items). Standardized answer options are provided and each question is rated on a scale from 0 to 4. A normalized score (100 indicating no symptoms and 0 indicating extreme symptoms) is then calculated for each subscale. The format is user-friendly and the questionnaire takes about 10 minutes to complete. The KOOS questionnaire is self-explanatory and can be administrated in the waiting room or used as a mailed survey [7]. The KOOS questionnaire includes the WOMAC Osteoarthritis Index LK 3.0 [2,3] in its complete and original format (with permission), and WOMAC scores can be calculated. The WOMAC is worldwide used in elderly subjects with knee or hip OA [2]. The Dutch version of the WOMAC is validated for hip OA patients [15].

##### Short Form-36 (SF-36)

The SF-36 is a generic health status questionnaire that contains 36 items. It measures eight dimensions (bodily pain; physical function; social function; role limitations because of physical problems; role limitations because of emotional problems; mental health; vitality; general health perceptions) and is widely used, has shown to be reliable and valid in the Dutch general population, and is easy to complete [1,16].

##### Visual analogue scale for pain

The Visual Analogue Scale (VAS) for pain is a simple way of measuring the intensity of pain. The 100-mm VAS is a unidimensional scale that is versatile, easy to use, and has been adopted in many settings. It has shown to be valid and reliable [13].

#### Statistical analysis

##### Internal consistency

A high degree of homogeneity is desirable in a scale. This has two implications: 1) the items should be at least moderately correlated with each other, and 2) each item should correlate above 0.20 with the total scale score [14]. These two factors form the basis of the various tests of homogeneity or internal consistency of the scale. The internal consistency was determined by calculating Cronbach's alpha. The widely accepted cut-off is that Cronbach's alpha should be 0.70 or higher for a set of items to be considered a (sub) scale [14,17].

##### Factor analysis

Factor analysis is a technique designed to reveal whether or not the pattern of responses on a number of tests can be explained by a smaller number of underlying traits or factors, with each factor reflecting a different construct [14]. Streiner et al. noted that an absolute minimum of five subjects per variable is necessary, with the proviso that there are at least 100 subjects. Exploratory factor analyses were conducted on all KOOS items using principal component analyses (PCA) with varimax rotation on the combined study population, because all subgroups had a number lower than 100. We first extracted factors with eigenvalues greater than 1. Next, we carried out a forced five, four, three, two and one factor solution.

First, we identified the number of meaningful factors based on the Scree plot and on the interpretation of the factor solutions. Using the Scree plot, we looked for a break between the factors with relatively large eigenvalues and those with smaller eigenvalues. Factors that appeared before the break were assumed to be meaningful, and factors that appeared on the approximately horizontal line after the break were considered to account for only a trivial amount of variance and were therefore not considered meaningful. Second, we looked at the factor structure and factor loadings after varimax rotation. Items with a factor loading less than 0.50 on all factors could be considered for exclusion. In other words factor analysis was performed in order to determine whether the KOOS questionnaire actually consists of 5 subscales.

##### Reliability

Reliability involves the degree to which the results of measurement are consistent across repeated measurements [14]. To estimate the test-retest reliability of the Dutch KOOS subscales, we calculated intraclass correlation coefficients (ICCs) with a 95% confidence interval (95% CI). Due to practical problems we only assed the test-retest reliability at the mild and moderate OA group and the revision TKA group. We used the ICC two-way random effects model type agreement to measure the reliability [18]. The ICC is generally considered to be good at 0.70 and above [14]. The standard error of measurement (SEM) is a measure of the absolute measurement error of a score, expressed in the unit of measurement of the instrument [19]. The SEM was calculated as the square root of the sum of the between administration variance and the residual variance [20].

##### Validity

Validity is the degree to which an instrument measures the construct it is intended to measure. Because of the absence of a gold standard the validity was expressed in terms of construct validity, which concerns the extent to which a particular measure relates to other measures consistent with theoretically derived hypotheses for the constructs that are being measured [21]. The construct validity of the KOOS questionnaire was determined by comparing its results with the generic SF-36 and the VAS for pain.

Hypotheses were formulated about the expected magnitude and direction of relationships between the subscales of the KOOS questionnaire and the other instruments. The formulation of the hypotheses was based on the starting point that there is a clear distinction between the subscales of the KOOS questionnaire. We defined the construct validity of the KOOS questionnaire as good if ≥ 75% of the hypotheses could be confirmed [22], moderate in case of 50–75% confirmation, and low when under 50% of confirmation. To evaluate the construct validity of the Dutch KOOS version, Spearman's correlations were calculated.

We formulated four hypotheses about convergent relations between the KOOS questionnaire, SF-36 and VAS for pain. The correlation between KOOS Pain and SF-36 BP, between KOOS Pain and SF-36 PF, KOOS (all subscales) and VAS for Pain and KOOS ADL and SF-36 PF should be ≥ 0.60. We expected that KOOS Pain has a stronger correlation with SF-36 BP compared to the correlation with SF-36 PF. This difference should be at least 0.05 higher. We further expected that KOOS Pain has a stronger correlation with VAS for pain compared to the correlation of the other subscales of the KOOS with the VAS for Pain. This difference should be at least 0.05 higher. KOOS ADL was expected to have a 0.05 higher correlation with SF-36 PF compared to the correlation of the other subscales of the SF-36.

We formulated five hypotheses about divergent relations between all subscales of the KOOS questionnaire and SF-36 GH: with correlations of ≤ 0.30. All other correlations between the KOOS subscales and the SF-36 should be higher than 0.30 and lower than 0.60.

##### Floor and ceiling effects

The presence of floor and ceiling effects may influence the reliability, validity and responsiveness of an instrument. An intervention effect might be missed for people who occupy the maximum score. Floor and ceiling effects were considered present if more than 15% of the respondents achieved the highest or lowest possible score [22].

Data were analysed with SPSS statistical software version 10.1. The level of significance for all statistical procedures was p ≤ 0.05.

## Results

Table 1 presents the characteristics of five patient groups. The first patient group consisted of 36 patients with mild OA (response rate of 79%). All patients filled in the questionnaires for the cross-sectional validity. For the test-retest reliability 35 patients filled in the KOOS questionnaire twice. The second patient group consisted of 62 patients with moderate OA (response rate of 76%) who filled in the questionnaires for the cross-sectional validity. Of these patients 53 filled in the KOOS questionnaire twice for the test-retest reliability. The third patient group consisted of 47 patients with severe OA (response rate of 54%). The fourth group consisted of 63 TKA patients (response rate of 77%) and the fifth group of 54 patients with a revision of the TKA (response rate of 75%). These patients filled in all questionnaires for the cross-sectional validity and 47 patients filled in the KOOS questionnaire twice for the test-retest reliability.

**Table 1:** Characteristics of the five patient groups

	**Mild OA (n = 36)**	**Moderate OA (n = 62)**	**Severe OA (n = 47)**	**TKA (n = 63)**	**Revision of TKA (n = 54)**
**Age in years**	36 (27–50)	56 (27–72)	65 (42–81)	61(42–78)	77 (36–89)
**Gender, women %**	22	32	52	51	78
**VAS pain**	0.7 (0.0–6.7)	3.9 (0.0–10.0)	6.1 (1.0–10.0)	1 (0.0–9.4)	5.0 (0–10)
**KOOS**					
**Pain**	85.3 **± **18.5	62.9 **± **25.7	41.8 **± **18.9	70.1 **± **24.5	61.6 **± **23.4
**Symptoms**	78.6 **± **7.1	63.2 **± **24.4	46.4 **± **18.7	72.3 **± **18.5	64.7 **± **21.5
**ADL**	91.2 **± **15.1	69.3 **± **24.2	43.4 **± **19.4	72.8 **± **23.3	56.6 **± **21.9
**Sport/recreation**	71.0 **± **23.4	36.2 **± **32.0	29.1 **± **39.2	33.2 **± **24.1	26.8 **± **34.1
**QoL**	67.0 **± **21.6	44.6 **± **26.3	30.9 **± **25.5	52.2 **± **23.8	36.6 **± **26.6
					
**SF-36**					
**BP**	84.7 **± **19.7	63.6 **± **24.0	34.6 **± **22.1	70.7 **± **24.9	55.4 **± **27.2
**PF**	86.4 **± **17.2	61.1 **± **24.8	34.9 **± **20.6	61.7 **± **23.9	32.9 **± **24.4
**SF**	92.4 **± **10.5	82.8 **± **21.9	61.7 **± **28.1	81.8 **± **28.6	64.1 **± **29.0
**RF**	77.8 **± **38.2	64.8 **± **39.7	26.6 **± **34.7	57.4 **± **43.7	31.0 **± **38.8
**RE**	91.7 **± **25.7	87.4 **± **28.5	57.4 **± **44.3	69.7 **± **43.6	59.9 **± **42.6
**MH**	86.8 **± **13.4	79.6 **± **19.2	69.1 **± **20.0	74.6 **± **21.7	70.3 **± **20.7
**VT**	75.4 **± **13.4	68.9 **± **19.3	53.9 **± **19.3	66.5 **± **21.8	55.5 **± **19.1
**GH**	84.0 **± **12.8	65.0 **± **20.8	60.2 **± **23.0	66.8 **± **24.1	51.4 **± **21.3

Results are presented as median (range) or mean **± **SD. Abbreviations: OA; osteoarthritis, TKA; total knee arthroplasty, Revision of TKA; Revision of total knee arthroplasty; VAS, Visual Analogue Scale; BP, bodily pain; PF, physical function; SF, social function; RF, role limitations because of physical problems; RE, role limitations because of emotional problems; MH, mental health; VT, vitality; GH, general health perception; ADL, Activities of daily living; QoL, Quality of life.

### Internal consistency

Table 2 presents the internal consistency expressed by Cronbach's alpha. For all patients groups Cronbach's alpha was for all subscales above 0.71, indicating a good internal consistency of all items in these scales and subscales. Except for the subscale Symptoms in the severe OA group a Cronbach's alpha of 0.56 was found, which indicates a moderate internal consistency.

**Table 2:** Internal consistency of the KOOS subscales, expressed by Cronbach's alpha

**KOOS subscales**	**Mild OA**	**Moderate OA**	**Severe OA**	**TKA**	**Revision of TKA**
**Pain **(9 items)	0.94	0.93	0.80	0.92	0.87
**Symptoms **(7 items)	0.71	0.83	0.56	0.74	0.78
**ADL **(17 items)	0.78	0.97	0.94	0.94	0.93
**Sport/Recreation **(5 items)	0.87	0.95	0.98	0.88	0.95
**QoL **(4 items)	0.81	0.85	0.73	0.81	0.90

Abbreviations: OA; osteoarthritis, TKA; total knee arthroplasty, Revision of TKA; Revision of total knee arthroplasty, ADL, Activities of daily living; QoL; Quality of life.

#### Factor analysis

The Scree plot showed a distinct break before factor 3, suggesting that only the first two factors were meaningful enough to be retained. This indicates that two factors may be adequate to describe the data. This initial solution accounted for 64% of the total variance for the Dutch version of the KOOS questionnaire (eigenvalue of 21.5 for the first factor and 3.7 for the second factor). However, in the two-factor solution, many items loaded on both factors. Therefore, we chose a forced one-factor solution, which accounted for 51.0% of the variance. The loading factors ranged from 0.37 – 0.85. The loading factor of the question S4 was lower than 0.40.

### Reliability

Table 3 presents the ICCs of all subscales of the KOOS questionnaire for patient groups with mild and moderate OA and after revision of the TKA patients. In these patient groups the ICCs were 0.70 or higher, indicating a good reliability. Only an ICC of 0.45 was found for the subscale Sport/recreation in the revision TKA group.

**Table 3:** Reliability of all subscales of the KOOS

		**Baseline mean (SD)**	**Retest mean (SD)**	**Change scores mean (SD)**	**SEM**	**ICC agreement**	**95% CI**
**Mild OA **(n = 35)	Pain	85.3 (18.5)	89.7 (12.5)	-4.4 (9.4)	7.2	0.80	0.60–0.90
	Symptoms	78.6 (7.1)	81.3 (16.8)	-2.2 (12.8)	9.0	0.74	0.54–0.86
	ADL	91.2 (15.1)	93.5 (10.8)	-2.3 (7.1)	5.2	0.85	0.71–0.92
	Sport/recreation	71.0 (23.4)	73.0 (22.9)	-1.7 (12.8)	9.0	0.85	0.73–0.92
	QoL	67.0 (21.6)	69.6 (20.5)	-2.5 (10.3)	7.4	0.88	0.77–0.94

**Moderate OA **(n = 53)	Pain	62.9 (25.7)	63.2 (23.7)	0.0 (12.9)	9.0	0.87	0.78–0.92
	Symptoms	63.2 (24.4)	66.2 (21.9)	-1.5 (11.3)	8.0	0.87	0.79–0.92
	ADL	69.3 (24.2)	69.2 (23.7)	0.6 (8.2)	5.8	0.94	0.90–0.97
	Sport/recreation	36.2 (32.0)	39.7 (32.5)	-4.8 (15.9)	11.6	0.87	0.78–0.92
	QoL	44.6 (26.3)	47.3 (25.4)	-2.1 (10.3)	7.4	0.91	0.86–0.95

**Revision of TKA **(n = 47)	Pain	61.6 (23.4)	63.9 (21.9)	-2.3 (14.3)	10.1	0.80	0.67–0.88
	Symptoms	64.7 (21.5)	62.9 (21.7)	1.7 (10.2)	7.2	0.89	0.81–0.94
	ADL	56.6 (21.9)	59.5 (22.9)	-2.9 (16.4)	11.7	0.73	0.56–0.83
	Sport/recreation	26.8 (34.1)	27.4 (34.2)	-2.2 (35.1)	24.6	0.45	0.19–0.66
	QoL	36.6 (26.6)	40.3 (27.5)	-3.7 (14.9)	10.8	0.84	0.73–0.91

Abbreviations: OA; osteoarthritis, SD, standard deviation; SEM, standard error of measurement; ICC agreement, intraclass correlation coefficient for agreement; CI, confidence interval. A normalized score (100 indicating no symptoms and 0 indicating extreme symptoms) is calculated for each subscale. Revision of TKA; Revision of total knee arthroplasty, AD; Activities of daily living; QoL; Quality of life.

The SEM ranged for the mild OA group between 5.2 and 9.0, for the moderate OA group between 5.8 and 11.6 and for patients after revision of the TKA between 7.2 and 24.6.

### Validity

Of the predefined hypotheses 60% or more could be confirmed for the study groups with mild OA and moderate OA and for the TKA patient population. For the severe OA group and the revision TKA group less than 45% could be confirmed. Tables 4, 5, 6, 7 and 8 show the correlations between the KOOS subscales, the SF-36 subscales and the VAS for pain. Overall, the highest correlations between the KOOS subscales and the SF-36 bodily pain and physical function were found. Correlations between the KOOS subscale Pain and the VAS-pain were between *r *= -0.28 and -0.79.

**Table 4:** Validity of the KOOS for the patient group with mild OA

	*KOOS Pain*	*KOOS Symptoms*	*KOOS ADL*	*KOOS Sport/Rec*	*KOOS QOL*
**SF-36 **Subscale					
BP	0.60*	0.44	**0.63**	0.41	0.38
PF	**0.64**	0.53	0.63*	0.46	0.56
SF	0.23	0.13	0.25	0.09	0.26
RF	0.28	0.37	0.35	0.36	0.40
RE	0.31	0.24	0.19	0.25	0.24
MH	0.42	0.20	0.42	0.30	0.38
VT	0.42	0.27	0.37	0.34	0.31
GH	*0.34*	*0.28*	*0.35*	*0.30*	*0.30*
**VAS for pain**	-0.79*	**-0.71**	**-0.78**	**-0.57**	**-0.59**

Validity is expressed by Spearman correlations between KOOS subscales, SF-36 subscales and VAS-pain. Abbreviations: OA; osteoarthritis, BP, bodily pain; PF, physical function; SF, social function; RF, role limitations because of physical problems; RE, role limitations because of emotional problems; MH, mental health; VT, vitality; GH, general health perception; ADL, Activities of daily living; QoL, Quality of life. In bold: convergente correlations that should be ≥ 0.60, In Italic: divergente correlations that should be ≤ 0.30. All other hypotheses are expected to be between 0.30 and 0.60. Correlations marked by * have to be 0.05 higher than the other convergent correlations.

**Table 5:** Validity of the KOOS for the patient group with moderate OA

	*KOOS Pain*	*KOOS Symptoms*	*KOOS ADL*	*KOOS Sport/Rec*	*KOOS QOL*
**SF-36 **Subscale					
BP	0.63*	0.57	**0.71**	0.43	0.62
PF	**0.72**	0.60	0.75*	0.60	0.70
SF	0.36	0.48	0.50	0.05	0.42
RF	0.23	0.32	0.36	0.12	0.26
RE	0.33	0.39	0.41	0.16	0.46
MH	0.23	0.36	0.27	-0.01	0.25
VT	0.37	0.35	0.48	0.05	0.37
GH	*0.32*	*0.22*	*0.32*	*0.13*	*0.29*
**VAS for pain**	-0.69*	**-0.57**	**-0.69**	**-0.38**	**-0.75**

Validity is expressed by Spearman correlations between KOOS subscales, SF-36 subscales and VAS-pain. Abbreviations: OA; osteoarthritis, BP, bodily pain; PF, physical function; SF, social function; RF, role limitations because of physical problems; RE, role limitations because of emotional problems; MH, mental health; VT, vitality; GH, general health perception; ADL, Activities of daily living; QoL, Quality of life. In bold: convergente correlations that should be ≥ 0.60, In Italic: divergente correlations that should be ≤ 0.30. All other hypotheses are expected to be between 0.30 and 0.60. Correlations marked by * have to be 0.05 higher than the other convergent correlations.

**Table 6:** Validity of the KOOS for the patient group with severe OA

	*KOOS Pain*	*KOOS Symptoms*	*KOOS ADL*	*KOOS Sport/Rec*	*KOOS QOL*
**SF-36 **Subscale					
BP	0.57*	0.55	**0.72**	0.37	0.47
PF	**0.36**	0.27	0.54*	0.12	0.22
SF	0.36	0.42	0.33	0.12	0.49
RF	0.28	0.21	0.37	0.04	0.32
RE	0.13	0.18	0.28	-0.04	0.25
MH	0.11	0.12	0.36	-0.07	0.18
VT	0.26	0.24	0.46	0.10	0.29
GH	*0.14*	*0.03*	*0.21*	*-0.11*	*0.16*
**VAS for pain**	-0.28*	**-0.43**	**-0.29**	**-0.19**	**-0.19**

Validity is expressed by Spearman correlations between KOOS subscales, SF-36 subscales and VAS-pain. Abbreviations: OA; osteoarthritis, BP, bodily pain; PF, physical function; SF, social function; RF, role limitations because of physical problems; RE, role limitations because of emotional problems; MH, mental health; VT, vitality; GH, general health perception, ADL; Activities of daily living, QoL; Quality of life. In bold: convergente correlations that should be ≥ 0.60, In Italic: divergente correlations that should be ≤ 0.30. All other hypotheses are expected to be between 0.30 and 0.60. Correlations marked by * have to be 0.05 higher than the other convergent correlations.

**Table 7:** Validity of the KOOS for the patient group with a primary TKA

	*KOOS Pain*	*KOOS Symptoms*	*KOOS ADL*	*KOOS Sport/Rec*	*KOOS QOL*
**SF-36 **Subscale					
BP	0.62*	0.58	**0.72**	0.48	0.62
PF	**0.66**	0.56	0.83*	0.67	0.64
SF	0.39	0.35	0.48	0.26	0.51
RF	0.53	0.53	0.68	0.57	0.49
RE	0.22	0.27	0.39	0.37	0.23
MH	0.46	0.46	0.52	0.37	0.52
VT	0.61	0.48	0.52	0.31	0.49
GH	*0.43*	*0.41*	*0.49*	*0.31*	*0.55*
**VAS for pain**	-0.70*	**-0.58**	**-0.59**	**-0.51**	**-0.27**

Validity is expressed by Spearman correlations between KOOS subscales, SF-36 subscales and VAS-pain. Abbreviations: OA; osteoarthritis, BP, bodily pain; PF, physical function; SF, social function; RF, role limitations because of physical problems; RE, role limitations because of emotional problems; MH, mental health; VT, vitality; GH, general health perception; ADL, Activities of daily living; QoL, Quality of life. In bold: convergente correlations that should be ≥ 0.60, In Italic: divergente correlations that should be ≤ 0.30. All other hypotheses are expected to be between 0.30 and 0.60. Correlations marked by * have to be 0.05 higher than the other convergent correlations.

**Table 8:** Validity of the KOOS for the patient group with a revision of the TKA

	*KOOS Pain*	*KOOS Symptoms*	*KOOS ADL*	*KOOS Sport/Rec*	*KOOS QOL*
**SF-36 **Subscale					
BP	0.49*	0.39	**0.50**	0.30	0.54
PF	**0.26**	0.20	0.44*	0.32	0.36
SF	0.20	0.11	0.24	0.16	0.42
RF	0.12	0.18	0.14	0.24	0.26
RE	0.10	0.09	0.00	-0.01	0.15
MH	0.15	0.26	0.21	0.05	0.28
VT	0.15	0.28	0.18	0.05	0.39
GH	*0.09*	*0.27*	*0.24*	*0.22*	*0.33*
**VAS for pain**	-0.47*	**-0.47**	**-0.29**	**-0.30**	**-0.21**

Validity is expressed by Spearman correlations between KOOS subscales, SF-36 subscales and VAS-pain. Abbreviations: OA; osteoarthritis, BP, bodily pain; PF, physical function; SF, social function; RF, role limitations because of physical problems; RE, role limitations because of emotional problems; MH, mental health; VT, vitality; GH, general health perception; ADL, Activities of daily living; QoL, Quality of life. In bold: convergente correlations that should be ≥ 0.60, In Italic: divergente correlations that should be ≤ 0.30. All other hypotheses are expected to be between 0.30 and 0.60. Correlations marked by * have to be 0.05 higher than the other convergent correlations.

### Floor and ceiling effects

Neither floor effects (indicating worst possible score) nor ceiling effects (indicating best possible score) were found for the patients with moderate OA patients and the TKA patients (Table 9). Only ceiling effects were present in the mild OA group for the subscales Pain, Symptoms and ADL and for the subscale Sport/Recreation in the severe OA TKA group. Floor effects were found for the subscales Sport/Recreation and Qol in the severe OA and revision TKA.

**Table 9:** Percentage ceiling/floor effects of the KOOS (best possible score/worst possible score)

	**Pain**	**Symptoms**	**ADL**	**Sport/Recreation**	**QoL**
**Mild OA **(n = 36)	**28/**0	**22/**0	**42/**0	14/0	11/0
**Moderate OA **(n = 62)	13/2	3/0	8/0	10/11	5/2
**Severe OA **(n = 47)	0/2	0/2	0/0	**20/38**	0/**15**
**TKA **(n = 63)	10/0	3/0	7/0	0/7	6/3
**Revision of TKA **(n = 54)	2/0	4/0	2/0	6/**38**	4/4

Abbreviations: OA; osteoarthritis, Revision of TKA, Revision of total knee arthroplasty; ADL, Activities of daily living; QoL, Quality of life.

## Discussion

The results of this validation study of the Dutch KOOS questionnaire showed a good internal consistency for all study groups. Reliability was also good in the mild and moderate OA group and the revision TKA group. It was not assessed the patients with severe OA and patients with a TKA. The construct validity was moderate for the patient groups with mild and moderate OA and for TKA patients, and lower for the severe OA and revision TKA patients. Ceiling effects were present in the mild OA group and in the severe OA group. Floor effects were seen in the patient group with severe OA group and the revision TKA group.

In this validation study Cronbach's alphas were above 0.70 for almost all subscales in our patient groups. This indicates a good internal consistency, which is in line with the study of Roos et al. [6,8]. However, for the subscale Symptoms in the severe OA population we found a Cronbach's of 0.56, indicating a moderate internal consistency. Deleting one or more questions did not result in a higher internal consistency. Kessler et al. and Xie et al. also found a lower Cronbach's alpha (< 0.70) for this subscale in patients with OA of the knee [23,24].

In our study, factor analysis was performed on the whole study population and we found that all items of the Dutch version of the KOOS questionnaire loaded on one factor. Our results are in contrast with the conclusion of Roos et al. that the KOOS items loaded on five factors [6]. However, our findings are in line with Thumboo et al. and Faucher et al. who claimed that the subscales Pain and Physical function of the WOMAC loaded on the same factor [25-28]. In the present study, the factor loading of the question S4 (can you straighten your leg fully) was lower than 0.40 which suggests that this item might be excluded from the questionnaire. Despite our preliminary results indicating that the Dutch version of the KOOS questionnaire contains one single factor, we retained in our analyses the original subscales of the Swedish version of the KOOS questionnaire. However, based on our findings we recommend additional factor analyses on other data sets, before changing the number of subscales of the Dutch version of the KOOS questionnaire.

In the present study the test-retest reliability was good for the patient groups with mild OA (ICC 0.74–0.88), moderate OA (ICC 0.87–0.94) and patients after a revision TKA (ICC 0.73–0.89). A lower ICC (0.45) for patients after a revision TKA for the subscale Sport/recreation was found. When deleting all outliers the ICC is still smaller then 0.70 (ICC 0.62). It is plausible that for these older patients questions about sport and recreation are less relevant.

The construct validity of the KOOS questionnaire was determined by comparing the KOOS subscales with the subscales of the SF-36 and the VAS for pain. Correlations between subscales, which measure the same construct, were compared. In our study we found the highest correlations between the KOOS subscales and the SF-36 subscales which are intended to measure the same constructs. Within the TKA patient group we found some higher correlation coefficients compared to the study of Roos et al. (ADL vs PF r = 0.83 vs 0.48 and Pain vs PF r = 0.66 vs 0.19) [8]. The correlations we found within the severe OA patient group (ranging from r = 0.12 to 0.57) were lower than found by Xie et al. They found correlations between r = 0.37 and 0.65 for the English version and r = 0.24 and 0.64 for the Chinese version of the KOOS [24]. Kessler et al. compared the subscales of the KOOS with the SF-12 for the same population and found a low correlation between the subscale Symptoms and the SF-12 (r = 0.05); the other subscales showed correlations of 0.60 or higher [23].

By only reporting the correlations coefficients it is not clear whether the construct validity of a questionnaire is sufficient or not. Therefore Terwee et al. developed quality criteria for design, methods and outcomes of studies to compare the measurement properties of health status questionnaires [22]. These authors recommended assessing the construct validity by testing predefined hypotheses (e.g., about expected correlations between measures or expected differences in scores between 'known' groups). Without specific hypotheses there is a risk of bias, because retrospectively it is tempting to generate alternative explanations for low correlations instead of concluding that the questionnaire may not be valid. Terwee et al. give a positive rating for construct validity if hypotheses are specified in advance and at least 75% of the results are in correspondence with these hypotheses [22]. Our choice that convergent correlations should have a correlation coefficient of ≥ 0.60 and divergent correlations of ≤ 0.30 is arbitrary. However, there is no consensus in literature how to deal with this issue. From our pre-defined hypotheses 60% or more could be confirmed in both the mild and moderate OA group and in patients after a TKA (moderate construct validity). Less than 45% from our hypotheses could be confirmed for patients with severe OA and after a revision TKA (lower construct validity).

The formulation of the hypotheses was based on the starting point that there is a clear distinction between the subscales of the KOOS questionnaire. However, with factor analysis we found that all items of the Dutch version of the KOOS questionnaire seem to load on one factor. This may explain the overlap between the correlations of the different constructs of the KOOS questionnaire with the SF-36. This is most obvious for the subscales Pain and ADL of the KOOS in relation to the subscales BP and PF of the SF-36. Previous studies showed that the WOMAC subscale pain and physical function loaded on the same factor [25-27]. Apparently it is difficult for patients to make a distinction between questions about pain and physical functioning in ADL. In our opinion this can be ascribed to the formulation of the questions; the term difficulty (translated in Dutch: 'moeite') may be not clear for some patients. The meaning of this term should be clarified or re-formulated. This was also suggested by Stratford et al, and Terwee et al. [29,30].

Because it is known that clinimetric properties are variable in different study populations [14], it is recommended to validate a questionnaire in the target population. This study showed that the clinimetric properties of the Dutch version of the KOOS questionnaire differed between the 5 different patient groups, which confirms the above described recommendation. Additionally, in future validation studies of the KOOS questionnaire, it may be of interest to evaluate the validity of the Dutch KOOS questionnaire by comparing the subscales of the KOOS questionnaire with the Dutch Oxford 12-item knee questionnaire. This latter questionnaire was considered to be valid and reliable in patients with OA of the knee [31]; however, it was not validated when we started the present study.

We observed ceiling effects only in the mild OA patient group for the subscales Pain, Symptoms and ADL of the KOOS questionnaire. It is plausible that these patients have few complaints of their knee and have no or minor clinical signs of OA, which can explain the presence of ceiling effects in this group of patients. Floor effects were only found in the subscale Sport/recreation in the patients with severe OA and in patients after revision of the TKA. Roos et al. stated that questions about sport and recreation also are relevant for older patients [8]. However, this does not seem to apply for patients after revision of the TKA. Because of severity of the disease and/or higher age, it is plausible that these patients do not participate in sport and recreational activities. Dividing the revision population into those younger than 65 years and older than 65 years resulted in floor effects of over 50% in the older patients. Questions about sport may be more relevant to younger patients than to older patients. Because the KOOS questionnaire was originally developed for younger patients this finding is not surprising.

Our study is not without limitations. First, because the selection of patients in the present study only allows statements on the reliability and validity of the KOOS questionnaire in patients with different stages of OA and it's treatment. The questionnaire was not studied in patients after a menisectomie or an ACL reconstruction. The results of the present study could not be generalized to patients with an acute knee trauma.

Second, a measurement tool can also be used to monitor the efficacy of an intervention or the disease process of the patient. For this goal the tool needs to be sensitive to detect clinically relevant changes during a certain period of time (responsiveness). ICCs are strongly influenced by the heterogeneity of the study population.

The interpretation of the SEM, i.e. whether it should be regarded as a large or a small measurement error, depends on what changes are minimal important on the KOOS subscales. The smallest detectable change (defined as 1.96*√2*SEM) has to be smaller than the minimal important changes [20]. Future studies should look at what changes in scores on the KOOS subscales constitutes minimal important change. In addition, the responsiveness of the KOOS questionnaire needs to be evaluated in a future study.

## Conclusion

Based on these different clinimetric properties within the present study we conclude that the KOOS questionnaire seems to be suitable for patients with mild and moderate OA and for patients with a primary TKA. The Dutch version of the KOOS had a lower construct validity for patients with severe OA on a waiting list for TKA and patients after revision of a TKA. However, the construct validity is only assessed by comparing it with the SF-36 and the VAS for pain, not with a knee specific questionnaire. Further validation studies on the Dutch version of the KOOS should include knee specific questionnaires for assessing the construct validity.

## Competing interests

The author(s) declare that they have no competing interests.

## Authors' contributions

IBG, MMF, CBT and MR designed the study. IBG wrote the manuscript and guided data collection, analyses and interpretation. MMF assisted with writing the manuscript, data collection, analyses and interpretation. CBT critically reviewed the manuscript and provided input for additional analyses and for the interpretation. MR and JANV critically reviewed the manuscript. All authors have read and approved the final manuscript.
